# Association between triglyceride-cholesterol-body weight index and sarcopenia in the older adults from China: A cross-sectional study

**DOI:** 10.1371/journal.pone.0342265

**Published:** 2026-02-04

**Authors:** Yanmei Ning, Doudou Li, Ting Zhang, Jiana Shi, Ying Hu

**Affiliations:** 1 School of Pharmacy, Hangzhou Normal University, Hangzhou, Zhejiang, China; 2 Clinical Pharmacy Center, Department of Pharmacy, Zhejiang Provincial People’s Hospital, Af-filiated People’s Hospital, Hangzhou Medical College, Hangzhou, China; University of Naples Federico II, ITALY

## Abstract

**Background:**

The triglyceride-cholesterol-body weight index (TCBI) is a simple and reliable indicator of nutritional status. This investigation aimed to explore the association between TCBI and sarcopenia among older individuals in China.

**Methods:**

The AWGS 2019 criteria were utilized to evaluate sarcopenia. TCBI was calculated as (TG × TC × BW)/1000. Logistic regression analysis explored the independent connection between TCBI and the occurrence of sarcopenia. RCS was employed to investigate both linear and nonlinear correlations, and threshold effects were scrutinized. Furthermore, subgroup and sensitivity analyses were also conducted.

**Results:**

This study included 5293 participants. The TCBI was found to be negatively associated with sarcopenia in a fully corrected model, with a 66% decrease in the prevalence of sarcopenia for each 1-unit increase in LgTCBI (OR = 0.34; 95% CI: 0.28–0.41; p < 0.001). The study showed that this association was further highlighted in those with normal BMI and remains consistent in sensitivity analyses. Furthermore, we found a nonlinear association between both TCBI and sarcopenia, with an inflection point of 7.05 by the RCS curve and threshold effect analysis.

**Conclusion:**

This study revealed a nonlinear negative correlation between TCBI and sarcopenia, highlighting the implications of these findings for the early screening and timely intervention of sarcopenia.

## Introduction

Sarcopenia is a progressive and generalized skeletal muscle disorder characterized by decreased muscle mass and impaired muscle function [1]. As the global population continues to age, sarcopenia has become an increasingly important public health issue. Its prevalence is rising steadily in older adults worldwide. Global studies estimate the prevalence of sarcopenia in individuals aged 60 and above ranges from approximately 10% to 27% [2]. In Asia, it ranges from 5.5% to 25.7% [3], with approximately 18.6% reported among the elderly in China [4]. The high prevalence of this condition is associated with numerous adverse outcomes, including an increased risk of falls, cognitive decline, muscle weakness, impaired physical function, and, in severe cases, mortality [5–7]. These consequences not only substantially diminish patients’ quality of life but also place a considerable burden on healthcare systems. Consequently, early screening and diagnosis of sarcopenia are essential to prevent physical functional decline and maintain quality of life in affected individuals.

At present, a growing number of studies have demonstrated that malnutrition is a risk factor for sarcopenia [8,9]. However, existing nutritional assessment tools, such as the Prognostic Nutritional Index (PNI) [10] and the Controlling Nutritional Status (CONUT) [11], have seen limited adoption in clinical practice due to their relative complexity. This highlights the need for simpler, more practical indicators to facilitate early nutritional risk screening. TCBI (triglycerides, total cholesterol, and body weight index), first introduced in 2018, is a simple calculated nutritional index [12]. Studies have already confirmed its value as a prognostic indicator for coronary artery disease [12], critical illness requiring mechanical circulatory support (MCS) devices [13], and heart failure [14]. Moreover, the TCBI also showed a negative correlation with stroke incidence [15], stroke-associated pneumonia (SAP) [16], and cognitive impairment [17].

Although the TCBI has been extensively studied in various diseases, its association with sarcopenia remains unexplored. Previous studies have suggested that higher levels of TG and TC may serve as protective factors against sarcopenia [18]. However, the relationship between sarcopenia and TCBI, which integrates triglycerides, total cholesterol, and body weight, remains to be fully clarified. Therefore, the present study aims to investigate the potential association between TCBI and sarcopenia, thereby providing a scientific foundation for optimizing screening protocols and formulating targeted intervention strategies.

## Methods

### Study population

The data utilized in this study were obtained from the China Health and Retirement Longitudinal Study (CHARLS), a nationally representative and ongoing longitudinal survey initiated in 2011. CHARLS employs a multistage, stratified, probability proportional to size (PPS) sampling strategy to recruit a representative cohort of Chinese residents aged 45 years and older. Data were collected through face-to-face interviews using standardized, structured questionnaires. The baseline survey encompassed 150 counties or districts and 450 villages or urban communities across 28 provinces in China. Follow-up interviews have been conducted biennially since the baseline wave. Ethical approval for the study was granted by the Biomedical Ethics Review Committee of Peking University (IRB00001052-11015). Written and oral informed consent was obtained from all participants before their participation. Comprehensive details regarding the study design and methodology of CHARLS have been documented in prior publications [19]. The data analyzed in this study were obtained from CHARLS and did not require additional ethical review by the investigator’s affiliated institution. Secondary analyses did not necessitate additional institutional review board approval.

**Fig 1:**
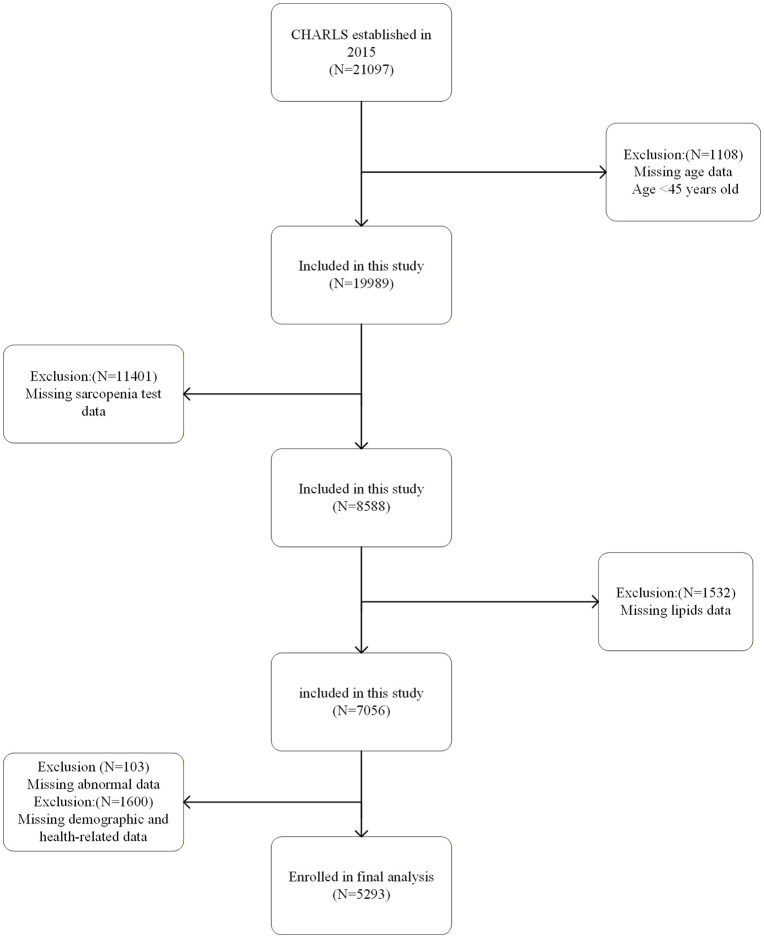
Flowchart of the population screening procedure.

This study conducted a cross-sectional analysis using data from wave 3 of the China Health and Retirement Longitudinal Study (CHARLS). Participants were excluded based on the following criteria: (1) age < 45 years; (2) missing data related to sarcopenia assessment; (3) missing lipids data;(4) abnormal data. After applying these exclusion criteria, a total of 5,293 eligible participants were included in the final analysis. Detailed inclusion and exclusion procedures are depicted in Fig 1.

### Assessment of sarcopenia

In this study, sarcopenia was assessed based on the criteria established by the Asian Working Group for Sarcopenia 2019 (AWGS 2019), which incorporates three key components: muscle strength, appendicular skeletal muscle mass (ASM), and physical performance [3]. Sarcopenia is identified in individuals with low muscle mass coupled with low muscle strength or low physical performance.

1) Handgrip strength was used as a measure of overall muscle strength. Measurements were conducted using a Yuejian TMWL-1000 dynamometer. Each participant performed two grip strength trials with each hand, and the highest value obtained from either hand was used for analysis. Low muscle strength was defined as a grip strength of less than 28 kg for males and less than 18 kg for females, in accordance with the AWGS 2019 criteria [3];
2) Muscle mass was estimated using anthropometric prediction equations that have been previously validated in Chinese populations. The equation used to estimate appendicular skeletal muscle mass (ASM) demonstrated strong concordance with measurements obtained by Dual X-ray absorptiometry (DXA) [20]:

$$\begin{array}{cc} \text{ASM}\;= & \;\text{0}.\text{193}\;*\;\text{Weight}\;(\text{kg})\;+\;\text{0}.\text{107}\;*\;\text{Height}\;(\text{cm})\;-\;\text{4}.\text{157}*\text{Gender}\;(\text{Males}\;=\;\text{1}\;\text{and Females}\;=\;\text{2})\;\\ & -\;\text{0}.\text{037}*\text{Age}\;(\text{years})\;\text{2}.\text{631} \end{array}$$

Referring to previous studies [21,22], the cut-off low muscle mass is set at the lowest 20% of sex-specific height-adjusted muscle mass (ASM/Ht^2^) in the study population, which is  < 5.27 kg/m^2^ for females and < 7.01 kg/m^2^ for males.

3) Due to the unavailability of Short Physical Performance Battery (SPPB) data for the participants, physical performance was assessed using gait speed and the five-time chair stand test as alternative measures. For gait speed assessment, each participant was asked to walk a 2.5-meter path at a normal pace, back and forth twice, with the total time recorded. The five-time chair stand test evaluated the time required to rise from a chair with a seat height of 47 centimeters five times consecutively, without using the arms. In accordance with the AWGS 2019 consensus criteria, low physical performance was defined as a gait speed of less than 1.0 m/s over a 6-meter distance, or a completion time of 12 seconds or more for the five-time chair stand test [3].

### Definition of TCBI

Venous blood samples were collected from participants in the CHARLS study, centrifuged, and then transported to the Chinese Center for Disease Control and Prevention (China CDC) in Beijing. All specimens were stored at −80°C before analysis. Biochemical assays were conducted at the Clinical Laboratory Center of Capital Medical University (CMU). Serum lipid profiles, including total cholesterol (TC) and triglycerides (TG), were measured using standardized enzymatic colorimetric methods. TCBI formula is as follows:


TCBI=TG(mg/dl)×TC(mg/dl)×BW(kg)/1000.

### Covariates

The main confounding factors were demographic and health-related covariates. Demographic variables included age, gender, residence(urban or rural), education (elementary school below or elementary school and above), and marital status. Health-related factors comprised smoking and drinking status (never or current), night sleep duration(<6 hours or ≥6 hours), falls (yes or no), BMI (underweight [<18.5 kg/m^2^], normal [18.5–23.9 kg/m^2^], overweight [≥24 kg/m^2^]), diabetes, hypertension, dyslipidemia, and biochemical markers such as (blood urea nitrogen [BUN], uric acid [UA], high-density lipoprotein cholesterol [HDL-C], low-density lipoprotein cholesterol [LDL-C], and C-reactive protein [CRP]).

### Statistical analysis

Counts and percentages(%) were used to express categorical variables and compared using the chi-square test. Mean ± SD or median(IQR) was used to represent continuous variables. Normality was assessed using the Shapiro-Wilk test. For group comparisons, one-way analysis of variance (ANOVA) or the Kruskal–Wallis H test, depending on the data distribution. Due to the skewed distributions of the TCBI, a logarithmic transformation (log) was performed to approximate normality.

We conducted univariate logistic regression analyses to investigate the relationship between each clinical variable and sarcopenia. Next, we used a multivariate logistic regression model, adjusting for age, sex, residence, education, marital status, smoking status, drinking status, BMI, night sleep duration, falls, diabetes, hypertension, dyslipidemia, BUN, LDL-C, HDL-C, CRP, and UA, to examine the independent association between the TCBI and sarcopenia. Subgroup and interaction analyses were carried out to explore potential effect modifications and to evaluate heterogeneity across different subgroups. Restricted cubic spline (RCS) curves were utilized to assess potential nonlinear relationships between TCBI and the risk of sarcopenia, along with a threshold effect analysis. Given that hypertension and dyslipidemia may also act as mediators, we carefully considered these factors when constructing the adjusted models. To minimize bias from omitted confounders, we comprehensively adjusted for these variables in the multivariate regression model. Additionally, separate mediation analyses were conducted for hypertension and dyslipidemia. Lastly, two sensitivity analyses were performed to assess the potential impact of over-adjustment on the results. All statistical analyses were conducted using RStudio (version 4.4.2) and EmpowerStats (version 4.2). A two-sided p-value < 0.05 was considered statistically significant.

## Results

### Clinical characteristics based on TCBI quartiles

The baseline characteristics of the study participants are summarized in Table 1.

**Table 1:** Baseline population characteristics of the cross-sectional study across to quartiles of TCBI.

Characteristics	TCBI quartiles				P
	Q1	Q2	Q3	Q4	
N	1324	1323	1323	1323	
Age(mean±SD,years)	68.6 (6.7)	67.7 (6.5)	67.2 (6.0)	66.4 (5.7)	<0.001
Gender,n(%)					<0.001
Male	832 (62.8%)	688 (52.0%)	616 (46.6%)	563 (42.6%)	
Female	492 (37.2%)	635 (48.0%)	707 (53.4%)	760 (57.4%)	
Marital status,n(%)					0.001
Married	1051 (79.4%)	1057 (79.9%)	1084 (81.9%)	1121 (84.7%)	
Single	273 (20.6%)	266 (20.1%)	239 (18.1%)	202 (15.3%)	
Residence,n(%)					<0.001
Rural	967 (73.0%)	885 (66.9%)	796 (60.2%)	732 (55.3%)	
Urban	357 (27.0%)	438 (33.1%)	527 (39.8%)	591 (44.7%)	
Education level,n(%)					0.014
Elementary school below	768 (58.0%)	745 (56.3%)	715 (54.0%)	690 (52.2%)	
Elementary school or above	556 (42.0%)	578 (43.7%)	608 (46.0%)	633 (47.8%)	
Drinking status,n(%)					<0.001
Never	615 (46.5%)	669 (50.6%)	728 (55.0%)	748 (56.5%)	
Drinker	709 (53.5%)	654 (49.4%)	595 (45.0%)	575 (43.5%)	
Smoking status,n(%)					<0.001
Never	568 (42.9%)	675 (51.0%)	738 (55.8%)	764 (57.7%)	
Smoker	756 (57.1%)	648 (49.0%)	585 (44.2%)	559 (42.3%)	
Fall down					0.482
No	837 (63.2%)	853 (64.5%)	815 (61.6%)	828 (62.6%)	
Yes	487 (36.8%)	470 (35.5%)	508 (38.4%)	495 (37.4%)	
BMI,n(%)					<0.001
Underweight	255 (19.3%)	86 (6.50%)	32 (2.4%)	11 (0.8%)	
Normal	903 (68.2%)	798 (60.3%)	641 (48.5%)	407 (30.8%)	
Overweight	166 (12.5%)	439 (33.2%)	650 (49.1%)	905 (68.4%)	
Night sleep,n(%)					0.578
Adequate sleep	863 (65.2%)	877 (66.3%)	886 (67.0%)	895 (67.6%)	
Sleep debt	461 (34.8%)	446 (33.7%)	437 (33.0%)	428 (32.4%)	
Dyslipidemia,n(%)					<0.001
No	1180 (89.1%)	1114 (84.2%)	1035 (78.2%)	891 (67.3%)	
Yes	144 (10.9%)	209 (15.8%)	288 (21.8%)	432 (32.7%)	
Diabetes,n(%)					<0.001
No	1244 (94.0%)	1207 (91.2%)	1170 (88.4%)	1086 (82.1%)	
Yes	80 (6.0%)	116 (8.8%)	153 (11.6%)	237 (17.9%)	
Hypertension,n(%)					<0.001
No	958 (72.4%)	842 (63.6%)	786 (59.4%)	632 (47.8%)	
Yes	366 (27.6%)	481 (36.4%)	537 (40.6%)	691 (52.2%)	
UA(mean±SD,mg/dl)	4.7 (1.4)	4.9 (1.4)	5.1 (1.4)	5.4 (1.4)	<0.001
HDL-C(mean±SD,mg/dl)	55.6 (13.0)	53.7 (12.6)	51.0 (11.2)	47.1 (9.7)	<0.001
LDL-C(mean±SD,mg/dl)	86.8 (21.9)	105.0 (24.4)	111.0 (27.8)	112.0 (32.6)	<0.001
CRP(IQR,mg/l)	1.0 [0.5; 2.1]	1.1 [0.7; 2.1]	1.4 [0.9; 2.5]	2.3 [1.4; 3.8]	<0.001
BUN(IQR,mg/dl)	16.0 [13.2; 19.3]	15.4 [12.9; 18.8]	15.4 [12.9; 18.5]	15.1 [12.6; 18.2]	<0.001
TC(mean±SD,mg/dl)	160 (27.8)	181 (29.4)	191 (32.0)	209 (38.2)	<0.001
TG(IQR,mg/dl)	68.1 [58.4; 78.8]	94.7 [84.1; 108.0]	131.9 [113.3; 152.7]	213.3 [172.6; 282.7]	<0.001
Sarcopenia,n(%)					<0.001
No	633 (47.8%)	966 (73.0%)	1108 (83.7%)	1238 (93.6%)	
Yes	691 (52.2%)	357 (27.0%)	215 (16.3%)	84 (6.4%)	

BMI, body mass index; UA, uric acid; HDL-C, high-density lipoprotein cholesterol; LDL-C, low-density lipoprotein cholesterol; CRP, C-reactive protein; BUN, blood urea nitrogen; TC, total cholesterol; TG, triglycerides. TCBI, triglycerides, total cholesterol, and body weight index. TCBI was classified as Q1(5.19–6.62), Q2 (6.62–7.06), Q3 (7.06–7.54), and Q4 (7.54–9.52).

The average age was 67.5 ± 6.3 years, with females accounting for 49.1% of the sample. After applying log-transformation to the TCBI, we divided the participants into four groups: Q1 (5.19–6.62), Q2 (6.62–7.06), Q3 (7.06–7.54), and Q4 (7.54–9.52). No significant differences across TCBI quartiles were observed for fall history or sleep duration (all p > 0.05). Compared with lower quartiles (Q1-Q3), Q4 participants were younger, more frequently female, married, living in rural areas, and less educated. They had higher proportions of never-smokers/never-drinkers, overweight status, diabetes, and hypertension. Participants have a higher level of UA, LDL-C, CRP, TC, and TG, but lower HDL-C and BUN (p < 0.05). Notably, the prevalence of sarcopenia was significantly lower (p < 0.05).

### Association between clinical characteristics and sarcopenia

Table 2 shows the outcomes of our univariate logistic regression analysis, which revealed that gender, drinking status, and falls were not significantly associated with sarcopenia (P > 0.05). Age, unmarried status, low educational level, current smoking, low body mass, insufficient sleep duration, HDL-C, LDL-C, CRP, and BUN levels were positively associated with the prevalence of sarcopenia (P < 0.05). Conversely, urban residency, overweight status, the presence of dyslipidemia, diabetes, hypertension, UA levels, and TCBI were negatively associated with sarcopenia risk (P < 0.05).

**Table 2:** The correlation between clinical characteristics and sarcopenia.

Characteristics	Statistics	OR (95% CI)	p
Age(mean±SD,years)	67.5 (6.3)	1.11 (1.10, 1.12)	<0.001
Gender,n(%)			
Male	2699 (51.0%)	Ref.	Ref.
Female	2594 (49.0%)	1.03 (0.91, 1.17]	0.597
Marital status,n(%)			
Married	4313 (81.5%)	Ref.	Ref.
Single	980 (18.5%)	1.64 (1.41, 1.91)	<0.001
Residence,n(%)			
Rural	3380 (63.9%)	Ref.	Ref.
Urban	1913 (36.1%)	0.49 (0.43, 0.56]	<0.001
Education level,n(%)			
Elementary school or above	2375 (44.9%)	Ref.	Ref.
Elementary school below	2918 (55.1%)	1.55 (1.36, 1.75)	<0.001
Drinking status,n(%)			
Never	2760 (52.1%)	Ref.	Ref.
Drinker	2533 (47.9%)	0.92 (0.81, 1.04]	0.183
Smoking status,n(%)			
Never	2745 (51.9%)	Ref.	Ref.
Smoker	2548 (48.1%)	1.15 (1.02, 1.31)	0.023
Fall down,n(%)			
No	3333 (63.0%)	Ref.	Ref.
Yes	1960 (37.0%)	1.09 (0.96, 1.24]	0.174
BMI,n(%)			
Normal weight	2749 (52.0%)	Ref.	Ref.
Overweight	2160 (40.7%)	0.01 (0.00, 0.01)	<0.001
Underweight	384 (7.3%)	16.00 (11.50, 22.90)	<0.001
Night sleep,n(%)			
Adequate sleep	3521 (66.5%)	Ref.	Ref.
Sleep debt	1772 (33.5%)	1.28 (1.13, 1.46)	<0.001
Hypertension,n(%)			
No	3218 (60.8%)	Ref.	Ref.
Yes	2075 (39.2%)	0.48 (0.42, 0.55)	<0.001
Dyslipidemia,n(%)			
No	4220 (79.7%)	Ref.	Ref.
Yes	1073 (20.3%)	0.38 (0.31, 0.46]	<0.001
Diabetes,n(%)			
No	4707 (88.9%)	Ref.	Ref.
Yes	586 (11.1%)	0.40 (0.31, 0.51)	<0.001
UA(mean±SD,mg/dl)	5.0 (1.4)	0.84 (0.80, 0.88)	<0.001
HDL-C(mean±SD,mg/dl)	51.8 (12.1)	1.04 (1.03, 1.05)	<0.001
LDL-C (mean±SD,mg/dl)	104 (28.8)	1.00 (0.99, 1.00)	<0.001
CRP (IQR,mg/l)	1.4 [0.8; 2.7]	1.01 (1.00, 1.02)	0.015
BUN (IQR,mg/dl)	15.4 [12.9; 18.8]	1.03 (1.01, 1.04)	0.001
TCBI^a^	7.1 (0.7)	0.23 (0.20, 0.26)	<0.001

CI, confidence interval; OR, odds ratio; BMI, body mass index; UA, uric acid; HDL-C, high-density lipoprotein cholesterol; LDL-C, low-density lipoprotein cholesterol; CRP, C-reactive protein; BUN, blood urea nitrogen; TC, total cholesterol; TG, triglycerides.

TCBI, triglycerides, total cholesterol, and body weight index.

^a^ The TCBI value underwent a log transformation in univariate analysis.

### Association between TCBI and sarcopenia

**Fig 2:**
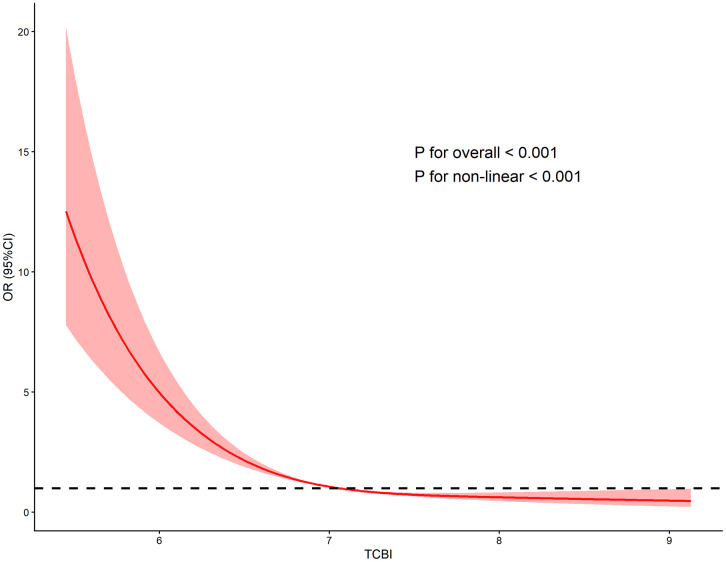
RCS curves are shown to reflect the dose-response association between TCBI and sarcopenia. The relationship was detected after adjusting for age, gender, education, residence, marital status, smoking status, drinking status, BMI, falls, night sleep duration, diabetes, hypertension, UA, HDL-C, LDL-C, CRP, BUN. The TCBI value underwent a log transformation.

Table 3 details the results of our multivariate logistic regression analysis. Following full multivariable adjustment(Model 3), each unit increase in LgTCBI was associated with a 66% decreased risk of sarcopenia (OR = 0.34, 95% CI: 0.28–0.41; p < 0.001). When LgTCBI as a categorical variable, in Model 3, the risk of sarcopenia in the Q2, Q3, and Q4 groups were reduced by 55% (OR = 0.45, 95% CI: 0.36–0.56; p < 0.001), 67%(OR = 0.33, 95% CI: 0.26–0.43; p < 0.001) and 79% (OR = 0.21, 95% CI: 0.15–0.29; p < 0.001) than the Q1 group. Additionally, RCS analysis demonstrated a L-shaped curve for the association between TCBI and sarcopenia (nonlinearity P < 0.001) (Fig 2). Threshold analysis identified an inflection point at approximately 7.05 for TCBI (p for likelihood test < 0.001). Above this threshold (TCBI > 7.05), each unit increase in TCBI was associated with a 41% decrease in sarcopenia risk [OR = 0.59, 95% CI: 0.38–0.91, p = 0.016]. In contrast, below the threshold (TCBI < 7.05), each unit increase was linked to an 81% reduction in risk [OR = 0.19, 95% CI: 0.13–0.27,p < 0.001]. (Table 4).

**Table 3:** The association between TCBI and sarcopenia.

TCBI^a^	OR (95% CI), p		
	Model 1	Model 2	Model 3
TCBI	0.18 (0.16, 0.20) <0.001	0.35 (0.30, 0.41) <0.001	0.34 (0.28, 0.41) <0.001
TCBI quartile(range)			
Q1(5.19–6.62)	Ref.	Ref.	Ref.
Q2(6.62–7.06)	0.34 (0.29, 0.40) <0.001	0.46 (0.38, 0.57) <0.001	0.45 (0.36, 0.56) <0.001
Q3(7.06–7.54)	0.18 (0.15, 0.22) <0.001	0.34 (0.27, 0.43) <0.001	0.33 (0.26, 0.43) <0.001
Q4(7.54–9.52)	0.06 (0.05, 0.08) <0.001	0.20 (0.14, 0.26) <0.001	0.21 (0.15, 0.29) <0.001

CI, confidence interval; OR, odds ratio.

Model 1:no covariates were adjusted.

Model 2: age, gender, education, residence, marital status, smoking status, drinking status, BMI, falls, night sleep duration were adjusted.

Model 3: age, gender, education, residence, marital status, smoking status, drinking status, BMI, falls, night sleep duration, diabetes, hypertension, dyslipidemia, UA, HDL-C, LDL-C, CRP, BUN were adjusted.

^a^The TCBI value underwent a log transformation.

**Table 4:** Threshold effect analysis of the relationship of TCBI with sarcopenia.

TCBI^a^	Adjusted OR (95%CI)	p
Model 1 fitting model by standard linear regression	0.33 (0.28, 0.41)	<0.001
Model 2 fitting model by two-piecewise logistic regression		
Inflection point	7.05	
<7.05	0.19 (0.13, 0.27)	<0.001
>7.05	0.59 (0.38, 0.91)	0.016
p for likelihood test		<0.001

CI, confidence interval; OR, odds ratio. Adjusted for age, gender, education, residence, marital status, smoking status, drinking status, BMI, falls, night sleep duration, diabetes, hypertension, dyslipidemia, UA, HDL-C, LDL-C, CRP, BUN.

^a^The TCBI value underwent a log transformation.

### Mediation effect of hypertension and dyslipidemia on the association between TCBI and sarcopenia

S1 Table shows the mediation analysis results of hypertension and dyslipidemia on the relationship between TCBI and sarcopenia. We found that the mediation proportions for hypertension and dyslipidemia were 2.5% and 1.9%, respectively. This suggests that both factors play a relatively weak mediating role in the association between TCBI and sarcopenia risk.

### Subgroups analyses

**Fig 3:**
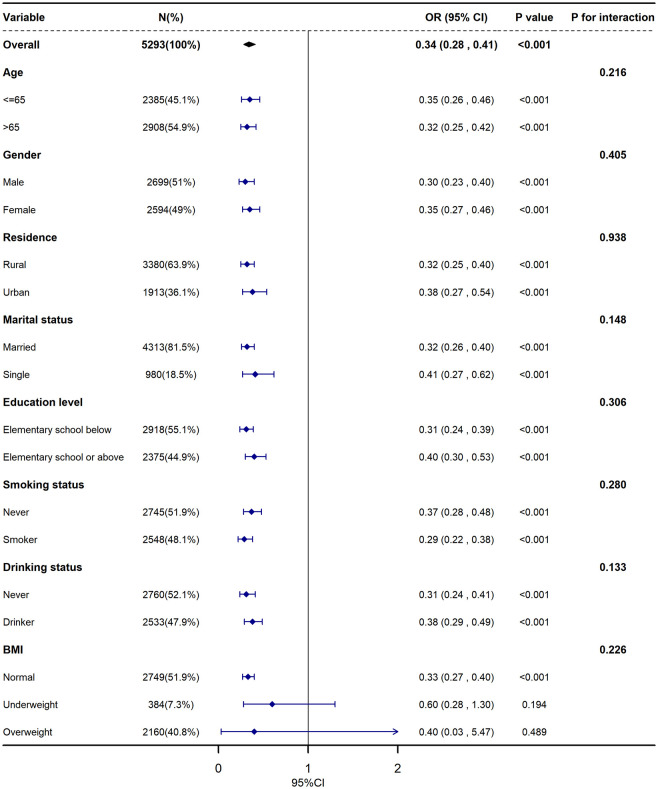
Subgroup analysis and forestplots of the association between TCBI and sarcopenia. CI, confidence interval; OR, odds ratio. This model was adjusted for age, gender, education, residence, marital status, smoking status, drinking status, BMI, falls, night sleep duration, diabetes, hypertension, UA, HDL-C, LDL-C, CRP, BUN.

Subgroup and stratified analyses were conducted by age, gender, residence, marital status, education level, smoking/drinking status, and BMI to evaluate effect modification across different populations and subgroups, as presented in Fig 3. The results showed no significant interactions among these subgroups, suggesting a consistent protective effect of TCBI against sarcopenia across all populations examined. Interestingly, the protective effect of TCBI on sarcopenia was found to be stronger among participants with a normal BMI.

### Sensitivity analyses

To assess the robustness of the study findings, two sensitivity analyses were conducted.

First, after individuals with dyslipidemia were excluded, each 1-unit increase in TCBI (LgTCBI) was associated with a 67% reduction in sarcopenia risk [OR = 0.33, 95% CI: 0.27–0.41, P < 0.001]. Furthermore, when LgTCBI was categorical variable, the incidence of sarcopenia in Q2, Q3, and Q4 groups was reduced by 5% (OR = 0.45,95% CI: 0.35–0.56, p < 0.001),75% (OR = 0.35,95% CI: 0.26–0.45, p < 0.001), and 80%(OR = 0.20,95% CI: 0.14–0.28, p < 0.001) than Q1 group (Table 5). Second, after not adjusting for hypertension and dyslipidemia, the association remained significant (S2 Table). Therefore, the negative relationship between the TCBI and sarcopenia risk is reliable and robust.

**Table 5:** Association between TCBI and sarcopenia in participants with normal blood lipids.

TCBI^a^	OR (95% CI), p
TCBI	0.33 (0.27, 0.41) <0.001
TCBI quartile(range)	
Q1(5.19–6.62)	Ref.
Q2(6.62–7.06)	0.45 (0.35, 0.56) <0.001
Q3(7.06–7.54)	0.35 (0.26, 0.45) <0.001
Q4(7.54–9.52)	0.20 (0.14, 0.28) <0.001

CI, confidence interval; OR, odds ratio. This model was adjusted for age, gender, education, residence, marital status, smoking status, drinking status, BMI, falls, night sleep duration, diabetes, hypertension, UA, HDL-C, LDL-C, CRP, BUN.

^a^The TCBI value underwent a log transformation.

## Discussion

This study identified a significant inverse relationship between the TCBI and the prevalence of sarcopenia in older adults in China, based on the China Health and Retirement Longitudinal Study (CHARLS) data. The association remained consistent after adjusting for potential confounders through multivariable logistic regression analyses. Although hypertension and dyslipidemia may partially mediate the relationship between the TCBI and sarcopenia, the mediation analysis in this study indicates a low proportion of mediation. Further subgroup and sensitivity analyses demonstrate that the primary findings remain robust. Furthermore, the research findings indicated a non-linear association between the TCBI and sarcopenia risk. Threshold effect analysis identified an inflection point around TCBI = 7.05, and an inverse association with sarcopenia risk was observed on both sides of this threshold. Notably, when the TCBI < 7.05, its protective effect against sarcopenia risk was relatively more pronounced.

Prokopidis, K. et al found that malnutrition was significantly associated with a high risk of sarcopenia, and that individuals with combined malnutrition and sarcopenia had a higher all-cause mortality rate [9]. Relying solely on albumin levels or body weight to predict a patient’s risk of sarcopenia is insufficient. Although nutrition screening tools like the NRS-2002 score or MNA-SF offer some predictive value, their implementation is complex and prone to subjective [23,24]. Consequently, these tools are not suitable for large-scale screening and early warning purposes. Past research has investigated the link between various lipid markers and sarcopenia, with findings suggesting that lower levels of TG and TC are associated with the onset of sarcopenia [18]. However, concrete data quantifying this relationship is scarce. To address this, we introduced the TCBI, a novel indicator that consolidates data from TC, TG, and body weight. This index was used to investigate the potential correlation between TCBI and the incidence of sarcopenia, aiming to provide a straightforward, quantitative, and comprehensive method for predicting sarcopenia risk. The findings of our investigation demonstrated a negative association between higher TCBI values and the risk of sarcopenia, irrespective of whether the TCBI is analyzed as a continuous variable or a categorical variable. This phenomenon can be explained by the concept of “reverse epidemiology.” Research indicates that lower levels of triglycerides and cholesterol may reflect a catabolic state or malnutrition, whereas higher body weight or elevated lipid levels in older adults may conversely be associated with better health outcomes. Consequently, higher TCBI values may indicate sufficient energy and nutritional reserves, a state potentially linked to reduced sarcopenia risk [25].

Meanwhile, the outcomes of the univariate logistic regression analysis revealed a negative correlation between hypertension, dyslipidemia, diabetes mellitus, and the risk of sarcopenia. These observations may be explained by the fact that individuals with hypertension or dyslipidemia frequently exhibit a higher intake of meat and animal-derived products, indicative of a superior overall nutritional status. This is commonly accompanied by an increased intake of calories and macronutrients, potentially leading to higher TCBI levels. Considering the inverse relationship between TCBI and sarcopenia, the higher nutritional intake in these groups may partially account for their decreased vulnerability to sarcopenia [26,27]. Our research also identified this correlation, particularly pronounced among individuals with normal weight. This insight will aid in the early screening of large populations, such as healthy individuals undergoing regular physical examinations. This group frequently omitted routine screenings due to the perception of a low risk of abnormal weight. Consequently, our findings have significant implications for diverse big data risk screening methodologies. These methods can facilitate early detection of sarcopenia, allowing for timely nutritional and exercise interventions to mitigate its prevalence in this population.

This study has several limitations. First, as data were obtained from the CHARLS database, key variables related to nutrition and sarcopenia were collected at fixed time points, which may not fully capture long-term nutritional status or changes in muscle mass. And much of the data was self-reported, which may introduce recall bias and limit the accuracy of the results. Second, due to its cross-sectional design, the study cannot establish a causal relationship between TCBI and sarcopenia; longitudinal studies are needed to confirm causality. Third, since the sample consisted solely of Chinese participants, the findings may not be generalizable to other populations due to potential genetic and lifestyle differences. Fourth, muscle mass was estimated using an anthropometric equation. While this equation has been validated in Chinese adults with results comparable to Dual X-ray absorptiometry (DXA) and Bioelectrical impedance analysis (BIA), it remains subject to measurement error [20,28]*.* Fifth, we assessed gait speed with a 2.5-m walk, rather than the 6-m walk recommended by the AWGS2019. Although a meta-analysis indicates that gait test distance does not affect the recorded speed, the estimates may still be subject to some bias [4,29].

## Conclusion

In conclusion, using data from the CHARLS survey, this study found that TCBI is non-linearly and negatively associated with the risk of sarcopenia, especially among individuals with normal BMI. The correlation appears to be more significant when the LgTCBI value is below 7.05.

## Supporting information

S1 Table Mediation analysis of hypertension and dyslipidemia on the association between TCBI and sarcopenia. (DOCX)

S2 Table The relationship between TCBI and sarcopenia without adjusting for hypertension and dyslipidemia. (DOCX)

S1 Data (XLSX)
