# Supplementary material for: Association between triglyceride-cholesterol-body weight index and sarcopenia in the older adults from China: A cross-sectional study
Source: PLoS One. 2026 Feb 4;21(2):e0342265. doi: 10.1371/journal.pone.0342265 (PMC12922771; doi:10.1371/journal.pone.0342265)
Supplement: S1 Table — (DOCX) [file pone.0342265.s001.docx]

S1 Table. Mediation analysis of hypertension and dyslipidemia on the association between TCBI and sarcopenia.

| **Mediator** | **Total effect** | **Mediation effect** | **Direct effect** | **PM(%)** | **P-value of PM** |
| --- | --- | --- | --- | --- | --- |
| Hypertension | − 0.060 (− 0.078,  − 0.031 ) | − 0.002 (− 0.003, − 0.001) | − 0.059 (− 0.077, − 0.031) | 2.5 | <0.001 |
| Dyslipidemia | − 0.060 (− 0.077, − 0.030) | − 0.001 (− 0.003, − 0.000) | − 0.057 (− 0.075, − 0.029) | 1.9 | 0.040 |

PM, proportion mediate.

Adjusting variables: age, gender, education, residence, marital status, smoking status, drinking status, BMI, falls, night sleep duration, diabetes, UA, HDL-C, LDL-C, CRP, BUN.

The TCBI is a continuous variable.
