# Supplementary material for: Association between triglyceride-cholesterol-body weight index and sarcopenia in the older adults from China: A cross-sectional study
Source: PLoS One. 2026 Feb 4;21(2):e0342265. doi: 10.1371/journal.pone.0342265 (PMC12922771; doi:10.1371/journal.pone.0342265)
Supplement: S2 Table — (DOCX) [file pone.0342265.s002.docx]

S2 Table. The relationship between TCBI and sarcopenia without adjusting for hypertension and dyslipidemia

| **TCBI^a^** | **OR (95% CI), p** |
| --- | --- |
| TCBI | 0.33 (0.27, 0.40) <0.001 |
| TCBI quartile(range) |  |
| Q1(5.19-6.62) | Ref |
| Q2(6.62-7.06) | 0.45 (0.36, 0.56) <0.001 |
| Q3(7.06-7.54) | 0.35 (0.25, 0.42) <0.001 |
| Q4(7.54-9.52) | 0.20 (0.14, 0.27) <0.001 |

CI, confidence interval; OR, odds ratio. This model was adjusted for age, gender, education, residence, marital status, smoking status, drinking status, BMI, falls, night sleep duration, diabetes, UA, HDL-C, LDL-C, CRP, BUN.

^a^The TCBI value underwent a log transformation.
